# Comparative detection of *Plasmodium vivax *and *Plasmodium falciparum *DNA in saliva and urine samples from symptomatic malaria patients in a low endemic area

**DOI:** 10.1186/1475-2875-9-72

**Published:** 2010-03-09

**Authors:** Pattakorn Buppan, Chaturong Putaporntip, Urassaya Pattanawong, Sunee Seethamchai, Somchai Jongwutiwes

**Affiliations:** 1 Molecular Biology of Malaria and Opportunistic Parasites Research Unit, Department of Parasitology, Faculty of Medicine, Chulalongkorn University, Bangkok, Thailand; 2 Department of Biology, Faculty of Science, Naresuan University, Phitsanulok Province, Thailand

## Abstract

**Background:**

Definite diagnosis of malaria relies on microscopy detection of blood stages of parasites in peripheral blood and requires blood sample collection. The nested PCR method has shown to be more sensitive and superior to microscopy in detecting co-infections of *Plasmodium *species in circulation while *Plasmodium falciparum *DNA can be identified in urine and saliva specimens of patients, albeit at a lower sensitivity.

**Methods:**

Matched blood, saliva and urine samples were collected from 100 microscopy-positive and 20 microscopy-negative febrile patients who attended a malaria clinic in Tak Province, northwestern Thailand for nested PCR analysis targeting the small subunit ribosomal RNA gene of human malaria. Both *P. falciparum *and *Plasmodium vivax *have been known to circulate at a comparable rate in the study area.

**Results:**

Comparing with microscopy results, nested PCR of saliva samples had a sensitivity of 74.1% for *P. falciparum *detection and 84% for *P. vivax *detection while 44.4% and 34.0% of the corresponding values were observed for urine samples. Both nested PCR results of saliva and urine samples had a specificity of 100% for identification of *P. falciparum *and *P. vivax *when compared with nested PCR results from blood. Co-infections of both species were found in four, 26 and 8 patients by microscopy and nested PCR of blood and saliva samples, respectively. Although the positive rates of nested PCR of saliva samples for *P. falciparum *increased with parasite density, no tendency occurred in results from nested PCR of saliva samples for *P. vivax *as well as those of urine samples.

**Conclusions:**

Saliva and urine samples could be alternative noninvasive sources of DNA for molecular detection of both *P. falciparum *and *P. vivax*. Further improvement of the detection method will offer an opportunity to use these samples for diagnosis of malaria.

## Background

*Plasmodium falciparum *and *Plasmodium vivax *are the main causative agents of human malaria accounting for 300-500 million cases and 130-145 million infections per annum, respectively [1,2]. Definite diagnosis of acute malaria infection in routine laboratory practice relies on demonstration of asexual erythrocytic stages from peripheral blood smears under light microscope. Although the method is feasible, economical and practical, a number of symptomatic cases have been left undiagnosed when parasitaemia did not reach the microscopic detection threshold. Hence, a more sensitive and specific PCR-based detection has been developed to amplify malarial DNA in patients' blood samples, unveiling a high prevalence of mixed species malaria infections in some endemic areas [3-5]. Failure to detect cryptic *P. falciparum *infections could lead to the risk of developing severe or fatal outcomes while missed vivax malaria may result in recurrent debilitating infections and economic loss [3]. Nevertheless, repeated examination of blood samples from malaria patients during post-treatment follow-up may at times result in poor compliance, especially among infants and young children. Therefore, an alternative means for a noninvasive malaria diagnosis is required.

Recent studies have shown that *P. falciparum *DNA could be detected in both urine and saliva samples of infected individuals in Zambia by means of PCR amplification of single copy genes encoding merozoite surface protein-2 and dihydrofolate reductase [6]. The sensitivity of PCR amplification of malarial DNA in these specimens seems to rely on patient's parasitaemia, DNA extraction method and length of target amplicon. A subsequent study in The Gambia has shown that detection of the small subunit ribosomal RNA gene (*SSU rRNA*) of *P. falciparum *in saliva and urine has high specificity comparable to that obtained from blood samples although the sensitivity of the method remains to be improved for useful diagnostic purposes [7]. Likewise, the sensitivity of PCR-based detection of the *P. falciparum *SSU rRNA gene using saliva samples reportedly increased to 82% when considering only isolates with a parasite density higher than or equal to 1,000 parasites/ml, the parasite level that usually found in most malaria patients in The Gambia and probably elsewhere [7]. Despite a lower sensitivity of PCR detection for *P. falciparum *from saliva and urine samples than that obtained from blood-derived DNA template, repeated noninvasive sample collections during drug trials or monitoring vaccine efficacy may be warranted [8].

Given that saliva and urine could be promising noninvasive sources of *P. falciparum *DNA for PCR diagnosis, it will be more beneficial when *P. vivax *DNA would also exist in these specimens. To further validate the potential roles of saliva and urine samples as the sources of *P. falciparum *DNA for PCR-based detection in a hypoendemic area outside Africa and to investigate the possibility of *P. vivax *detection in these samples, we performed a cross-sectional study in Thailand where both *Plasmodium *species contribute ~99% of all malaria cases [5,9,10].

## Methods

### Study area

This study was performed in a malaria clinic at Ta Song Yang District in Tak Province, ~550 km northwest of Bangkok, Thailand bordering Myanmar (GPS N17° 13' 36", E98° 13' 30"). Ta Song Yang District occupies 355 km^2 ^with a population density of ~24 persons/km^2^. Most of the areas are mountainous and filled with forests. In the rainy season, villagers would get into the jungle for a long period to cut the bamboo shoots for sale where they often return also with febrile malaria attack. Annual malaria transmission in Thailand follows a bimodal pattern, peaking in May-July and October-November [10]. The number of malaria cases in this country exhibits remarkable regional differences with the highest annual parasite incidence (API) of ~15.5 in Tak Province [9,10]. A comprehensive study of malaria species in this community comprised an almost equal distribution of *P. falciparum *and *P. vivax *while co-infection of both species accounted for ~24% of all malaria cases [5].

### Patients and sample collection

A cross-sectional study was conducted during June and July 2008. Inclusion criteria were febrile patients (oral temperature > 37.5°C) of any age group seen at Ta Song Yang malaria clinic who were willing to participate in this study during May and June 2008. Exclusion criteria were those having previous anti-malarial treatment or presence of clinical signs and symptoms of severe malaria [11]. In total, 100 malaria positive individuals were recruited in which diagnosis was initially performed by Giemsa-stained thick blood film at the malaria clinic. In addition, 20 other subjects were included who had febrile illness after entry and stay during the past one month in the forest area where malaria transmission was highly possible. Therefore, malaria positive rate in this study does not reflect the actual prevalence in the study population. Approximately 2 ml of venous blood were drawn from each subject after informed consent. Blood samples were preserved using EDTA as an anticoagulant and kept on ice during transportation from the study site to our laboratory at Chulalongkorn University where samples were subsequently stored at -40°C until use. Collection of urine and saliva samples was done immediately after blood sample collection and prior to anti-malarial treatment. Approximately 1 - 2 ml of saliva sample and 20 ml of midstream urine were obtained from each case. Half volume of these samples was kept on ice during transportation to the laboratory while the remaining part was preserved by adding ~two volumes of absolute ethanol and kept at ambient temperature (25°C - 35°C) until DNA extraction.

### Microscopy

Both thin and thick blood smears were prepared for each isolate and stained with 10% Giemsa solution. The thin blood film was examined for at least 200 microscopic fields and the thick blood film for at least 200 leukocytes, using a 100× objective. The microscopist who has more than 20 years of experience in identification of malaria and other human parasites was blinded to any clinical diagnosis, onsite microscopy-based detection and PCR results during the period of this study. The parasite density was estimated by assuming a leukocyte count of 7,000/ml [12].

### DNA extraction

DNA of each isolate was extracted from 200 ml of blood, saliva and urine samples using Qiagen DNA Mini Kit. DNA was purified following the manufacturer's recommendation.

### PCR-based detection

Detection of *P. falciparum *and *P. vivax *was done by nested PCR amplification protocol as described by Putaporntip *et al *[5]. Briefly, 2 ml of DNA was added to a total volume of 20 ml amplification reaction mixture with *Plasmodium *genus-specific outer primers derived from the SSU rRNA gene (M18SF0: 5'-CCATTAATCAAGAACGAAAGTTAAGG-3' and M18SR0: 5'-CAAGGAAGTTTAAGGCAACAACA-3') for primary PCR. Thirty-five cycles (94°C for 40 s, 60°C for 30 s and 72°C for 1 min) were performed. One ml of PCR product from primary PCR was used as DNA template for secondary PCR in which amplification for *P. falciparum *and *P. vivax *was done in separate reaction tubes. The amplification reaction and thermal cycling profile for secondary PCR were essentially as those for primary PCR except that respective pair of the species-specific inner primers (*P. falciparum*, PF18SF: 5'-CATCTTTCGAGGTGACTTTTAG-3' and PF18SR: 5'-GTTTTTTACTCTATTTCTCTCTTC-3'; *P. vivax*, PV18SF: 5'-GAATTTTCTCTTCGGAGTTTATTC-3' and PV18SR: 5'-TAACAGTTTCCCTTTCCCTTTTCTAC-3'; *P. malariae*, PM18SF: 5'-GAGACATTCATATATATGAGTG-3' and PM18SR: 5'-GTTTTTTTTAATAAAAACGTTCTTTTCCC-3'; *P. ovale*, PO18SF: 5'-GAAAAFFCCTTTTGGAAATTTCTTAG-3' and PO18SR: 5'-GATACATTATAGTGTCCTTTTCCC-3' and *P. knowlesi*, PK18SF: 5'-GAGTTTTTCTTTTCTCTCCGGAG-3' and PK18SR: 5'-ACGTTAAATGTGATTCCTTTCCC-3') and 25 cycles were used. The PCR products were separated in 1% and 2% agarose gels for primary and nested PCR, respectively. After staining with ethidium bromide, the gel was visualized under a UV light. The PCR amplified fragments of the SSU rRNA gene of *P. falciparum *and of *P. vivax *were 452 bp and 419 bp, respectively.

### Data analysis

Diagnostic performance for each test was evaluated with the results from nested PCR assay of DNA extracted from blood samples as the gold standard. Performance indices were the number of true positive (TP), number of true negative (TN), number of false positive (FP) and number of false negative (FN). Sensitivity was expressed as TP/(TP+FN) and specificity as TN/(TP+FP). Accuracy of the tests were calculated as (TP/TN)/number of all tests. *Kappa *statistics was used to compare the agreement against which might be expected by chance with possible values range from range from +1 (perfect agreement) via 0 (no agreement above that expected by chance) to -1 (complete disagreement) [13].

### Ethics

The ethical aspects of this study have been approved by the Institutional Review Board of Faculty of Medicine, Chulalongkorn University.

## Results

Of 120 febrile individuals participated in this study, 73 (60.8%) were males and 47 (39.2%) were females. The age range was 4 to 60 years old (mean = 20.3 years; median = 15.5 years). The majority of these subjects (82.5%) had experienced one or more episodes of previous malaria attack but not during the past one month prior to sample collection. Patients with microscopy positive for asexual blood stages of malaria had body temperature from 38°C to 41°C and none had overt severe manifestations.

Microscopic examination of blood films by the experienced microscopist has detected malaria parasites in 100 patients, which was in good agreement with the results obtained from microscopy detection by a local staff at the malaria clinic. Of these, perfect agreement was observed for single infection of *P. falciparum *in 50 patients and *P. vivax *in 46 patients while co-infections of both species were observed in four isolates by the experienced microscopist but not by the local malaria clinic staff. The overall parasite density of microscopy positive samples ranged from 35 to 311,395 parasites/ml (geometric mean = 13,920 parasites/ml). Isolates containing single infection of *P. falciparum *as determined by nested PCR had parasite density ranged from 35 to 217,805 parasites/ml (geometric mean = 2,761 parasites/ml) while that of *P. vivax *was between 35 to 44,520 parasites/ml (geometric mean = 1,248 parasites/ml).

The nested PCR method using DNA templates from blood samples has detected 106 febrile patients infected with *P. falciparum *(n = 43), *P. vivax *(n = 37) and co-infection of both species (n = 26). None gave positive tests for *Plasmodium malariae*, *Plasmodium ovale *and *Plasmodium knowlesi*-specific PCR primers. All 100 patients harboring malaria parasites in their blood smears gave positive results for PCR assay while six patients whose blood smears were negative by microscopy were positive for *P. falciparum *(n = 2), *P. vivax *(n = 1) and mixed infection of both species (n = 3) by PCR method (Table 1).

**Table 1:** Microscopy diagnosis and nested PCR detection of *Plasmodium falciparum *and *P. vivax *in blood, saliva and urine samples.

Method	Specimen (n = 120)	Result	Nested PCR (blood)				Total
			
			*P. falciparum*	*P. vivax*	*P. falciparum *+ *P. vivax*	Negative	
Microscopy	Blood	*P. falciparum*	41	0	9	0	50
		*P. vivax*	0	36	10	0	46
		*P. falciparum*+ *P. vivax*	0	0	4	0	4
		Negative	2	1	3	14	20
							
Nested PCR	Saliva (in ethanol)	*P. falciparum*	27	0	5	0	32
		*P. vivax*	0	32	5	0	37
		*P. falciparum*+ *P. vivax*	0	0	7	0	7
		Negative	16	5	9	14	44
							
	Saliva (on ice)	*P. falciparum*	18	0	5	0	23
		*P. vivax*	0	12	5	0	17
		*P. falciparum*+ *P. vivax*	0	0	3	0	3
		Negative	25	25	13	14	77
							
	Saliva (all)*	*P. falciparum*	31	0	6	0	37
		*P. vivax*	0	32	8	0	40
		*P. falciparum*+ *P. vivax*	0	0	8	0	8
		Negative	12	5	4	14	35
							
	Urine (in ethanol)	*P. falciparum*	16	0	4	0	20
		*P. vivax*	0	8	2	0	10
		*P. falciparum*+ *P. vivax*	0	0	0	0	0
		Negative	27	29	20	14	90
							
	Urine (on ice)	*P. falciparum*	8	0	2	0	10
		*P. vivax*	0	6	2	0	8
		*P. falciparum*+ *P. vivax*	0	0	0	0	0
		Negative	35	31	22	14	102
							
	Urine (all)*	*P. falciparum*	19	0	6	0	25
		*P. vivax*	0	13	4	0	17
		*P. falciparum*+ *P. vivax*	0	0	0	0	0
		Negative	24	24	16	14	78
							
		Total	43	37	26	14	120

* Positives from samples either kept on ice or preserved in ethanol.

The nested PCR assay has detected malaria SSU rRNA gene fragments in 43 of 120 saliva samples that were kept on ice during transportation. However, preservation of saliva samples in ethanol has increased more than twice the yield of positive results as 76 samples gave positive tests. Thirty-nine saliva samples preserved in ethanol gave positive results while the same samples kept on ice were negative and the opposite was true in nine isolates. In total, 85 saliva samples were positive by nested PCR method. Likewise, unpreserved urine samples showed a lower positive rate than those preserved in ethanol, i.e. 18 and 30 positives, respectively. Identification of malaria species by microscopy and nested PCR of blood, saliva and urine is shown in Table 1. Consistent with our previous study, co-infection of both *P. falciparum *and *P. vivax *accounted for 26% of all malaria positive cases from northwest Thailand as detected by nested PCR of blood [5]. Interestingly, nested PCR assay using saliva samples outperformed microscopy in detection of mixed species infection (Table 1). None of the isolates that were negative by PCR test using DNA template from blood (n = 14) gave positive results by microscopy or PCR assays of saliva and urine samples.

When *P. falciparum *and *P. vivax *were considered separately, nested PCR assay displayed more sensitivity of detection using DNA templates extracted from ethanol preserved saliva and urine samples than the unpreserved corresponding ones (Table 2). Evaluation of diagnostic performance using results from nested PCR of blood as gold standard has shown that sensitivity of microscopy for *P. falciparum *detection was 78.3% and *P. vivax *79.4%. PCR assays of ethanol-preserved saliva and urine specimens uniformly outperformed those kept on ice. In total, the sensitivity of combined results from saliva samples kept on ice and ethanol preserved ones was 65.2% for *P. falciparum *and 69.8% for *P. vivax*. Likewise, the sensitivity of combined results from urine samples preserved on ice and ethanol preserved ones was 36.2% and 27.0% for *P. falciparum *and *P. vivax*, respectively. It is noteworthy that combined PCR results of saliva kept on ice and those preserved in ethanol exhibited slightly lower accuracy and kappa values for *P. falciparum *and *P. vivax *than results from microscopy (Table 2).

**Table 2:** Diagnostic performance of microscopy and nested PCR assays using DNA templates from saliva and urine samples.

Method/Specimen	Category	% Sensitivity	% Specificity	Accuracy	*Kappa*
Microscopy	*P. falciparum*	78.3	100	87.5	0.75
	*P. vivax*	79.4	100	89.2	0.79
					
Nested PCR					
Saliva (in ethanol)	*P. falciparum*	56.5	100	75.0	0.53
	*P. vivax*	69.8	100	84.2	0.69
Saliva (on ice)	*P. falciparum*	37.7	100	64.2	0.34
	*P. vivax*	31.8	100	64.2	0.31
Saliva (all)	*P. falciparum*	65.2	100	80.0	0.61
	*P. vivax*	76.2	100	87.5	0.75
Urine (in ethanol)	*P. falciparum*	29.0	100	59.2	0.26
	*P. vivax*	15.9	100	55.8	0.15
Urine (on ice)	*P. falciparum*	14.5	100	50.8	0.13
	*P. vivax*	12.7	100	54.2	0.12
Urine (all)	*P. falciparum*	36.2	100	63.3	0.33
	*P. vivax*	27.0	100	61.7	0.26

Note. Nested PCR results from blood samples are used as the reference standard.

Using results from microscopy as reference, nested PCR assay of blood detected malaria in all microscopy positive samples and, additionally, *P. falciparum *in 22.7% and *P. vivax *in 18.6% of microscopy-negatives. PCR assay of saliva samples identified *P. falciparum *in 74.1% and *P. vivax *in 84.0% of microscopy-positives while five and six samples diagnosed to be negative by microscopy contained *P. falciparum *and *P. vivax *in their respective saliva samples. Meanwhile, 44.4% and 34.0% of microscopy positive isolates possessed *P. falciparum *and *P. vivax *DNA in their urine. In addition, one microscopy negative isolate was positive for *P. falciparum *in urine specimen (Table 3). Although the positive rates of nested PCR for *P. falciparum *detection in saliva exhibited some tendency toward increasing parasite density (*r *= 0.797, df = 3, p = 0.055), other tests did not show a similar trend (*r *= 0.459 - 0.745, p = 0.074 - 0.541) (Table 4).

**Table 3:** Comparison of nested PCR assays using blood, saliva and urine samples with microscopy results as reference.

	Microscopy (%)			
	
Nested PCR	*P. falciparum*		*P. vivax*	
		
	Positive (n = 54)	Negative (n = 66)	Positive (n = 50)	Negative (n = 70)
Blood				
Positive	54 (100)	15 (22.7)	50 (100)	13 (18.6)
Negative	0	51 (77.3)	0	57 (81.4)
Saliva (in ethanol)				
Positive	35 (64.8)	4 (6.1)	40 (80.0)	4 (5.7)
Negative	19 (35.2)	62 (93.9)	10 (20.0)	66 (94.3)
Saliva (on ice)				
Positive	24 (44.4)	2 (3.0)	18 (36.0)	2 (2.9)
Negative	30 (55.6)	64 (97.0)	32 (64.0)	68 (97.1)
Saliva (all)				
Positive	40 (74.1)	5 (7.6)	42 (84.0)	6 (8.6)
Negative	14 (25.9)	61 (92.4)	8 (16.0)	64 (91.4)
Urine (in ethanol)				
Positive	20 (37.0)	0	10 (20.0)	0
Negative	34 (63.0)	66 (100)	40 (80.0)	70 (100)
Urine (on ice)				
Positive	9 (16.7)	1 (1.5)	8 (16.0)	0
Negative	45 (83.3)	65 (98.5)	42 (84.0)	70 (100)
Urine (all)				
Positive	24 (44.4)	1 (1.5)	17 (34.0)	0
Negative	30 (55.6)	65 (98.5)	33 (66.0)	70 (100)

**Table 4:** Relationship between parasite density and positive tests by nested PCR of saliva and urine samples from malaria patients with single infections.

Specimen	Parasites/μl	No. positives/Total positives by blood PCR (%)	
		
		*P. falciparum*	*P. vivax*
Saliva			
	<1,000	5/7 (71.4)	2/3 (66.7)
	>1,000	25/34 (73.5)	30/33 (90.9)
	>5,000	23/27 (85.2)	15/16 (93.8)
	>10,000	18/19 (94.7)	11/12 (91.7)
	>50,000	8/8 (100)	-
Urine			
	<1,000	2/7 (28.6)	0/3 (0)
	>1,000	17/34 (50.0)	13/33 (39.4)
	>5,000	11/27 (37.0)	6/16 (37.5)
	>10,000	10/19 (52.6)	4/12 (33.3)
	>50,000	5/8 (62.5)	-

Note: Co-existence of both malaria species in three isolates was excluded from analysis.

## Discussion

Over the past decades, alternative tests for malaria diagnosis have been developed in order to increase the diagnostic performance of microscopy by species-specific PCR method, and to facilitate diagnosis by the advent of rapid diagnostic tests so that electricity and well-trained personnel are not required [14]. However, the pre-requisite for all these tests depends on blood samples containing parasite materials. Recent studies have shown that DNA of *P. falciparum *can be demonstrated in saliva and urine samples of infected individuals paving a novel alternative source of specimens for potential malaria diagnosis. Evaluation of PCR-based detection of *P. falciparum *in Gambian patients using saliva and urine samples compared with microscopy has revealed a high specificity (97%-98%) while its sensitivity remains moderate to low (73% for saliva and 32% for urine samples) [7].

Results from this study have reaffirmed that *P. falciparum *DNA could be identified in both saliva and urine samples of infected individuals in Tak Province. PCR assays of saliva and urine samples comparing with microscopy in this study had sensitivity of 74.1% and 44.4%, respectively, which are in good agreement with previous study by others using samples from patients in The Gambia where malaria transmission is much more intense than in Thailand [7]. It is, therefore, likely that excretion or transport of malarial DNA from circulation to saliva and urine occur similarly between these populations during the course of infection. Importantly, this study demonstrated for the first time that *P. vivax *can also be detected in both saliva and urine samples of patients at a comparable diagnostic performance for *P. falciparum*. Furthermore, co-infection of *P. falciparum *and *P. vivax *could be concordantly diagnosed in saliva and blood samples in 8 patients or 30.8% of mixed infections identified by PCR from blood samples.

Proper preservation of clinical specimens from the field to well-equipped laboratory is a key factor influencing the performance of the tests. Because malaria is endemic in tropical countries, trace amount of bacterial or other microorganism contaminants in urine and saliva samples could interfere with subsequent malarial DNA extraction. This study has shown that saliva and urine samples preserved in ethanol yielded positive results by nested PCR method superior to those kept on ice without preservation; thereby the less efficiency of PCR performance for samples kept on ice could reflect a remarkable loss of malarial DNA after prolonged storage without ethanol preservation. It seems likely that malarial DNA recovered from ethanol preserved saliva and urine samples in this study gave comparable PCR positive rate to those freshly collected from patients in The Gambia [7], suggesting that ethanol preservation is suitable for sample collection in field study.

The amount of *P. falciparum *DNA detected in urine and saliva samples has reportedly been ~2500-fold and ~600-fold less than that in blood concurrently obtained from infected individuals, a crucial issue that potentially compromises their diagnostic performance [7]. It is interesting to note that the sensitivity of PCR-based detection for *P. falciparum *in saliva comparing with microscopy of malaria patients in The Gambia seems to increase in samples with parasite density greater or equal to 1000 parasites/ml [7]. This study has shown that the positive rates of nested PCR for *P. falciparum *detection in saliva samples increased in isolates with high parasite density and such correlation was approaching significant level. On the other hand, no such trends were observed in urine samples for *P. falciparum *and both saliva and urine samples for *P. vivax*, suggesting that the presence of malarial DNA in urine and saliva may not directly correlate with concurrent parasite density in circulation. Further study is required to elucidate how malarial DNA is transported to saliva and urine of malaria patients. Nevertheless, improvement of detection method is of primary importance before saliva and urine samples can be reliably applied for alternative diagnosis of malaria parasites or evaluation of malaria control measures such as vaccine efficacy.

## Competing interests

The authors declare that they have no competing interests.

## Authors' contributions

CP and SJ involved in study design, supervised and managed the project. PB, CP, UP, SS and SJ carried out field work. PB and CP performed molecular assay. UP contributed microscopy-based detection and estimation of parasite density. PB, CP, SS and SJ analysed data. CP and SJ wrote the manuscript. All authors read and approved the final manuscript.

## References

[B1] Hay SI Guerra CA Tatem AJ Noor AM Snow RW The global distribution and population at risk of malaria: past, present, and future Lancet Infect Dis 2004 4 327 336 10.1016/S1473-3099(04)01043-6 15172341 PMC3145123

[B2] Snow RW Guerra CA Noor AM Myint HY Hay SI The global distribution of clinical episodes of *Plasmodium falciparum *malaria Nature 2005 434 214 217 10.1038/nature03342 15759000 PMC3128492

[B3] Mayxay M Pukrittayakamee S Newton PN White NJ Mixed-species malaria infections in humans Trends Parasitol 2004 20 233 240 10.1016/j.pt.2004.03.006 15105024

[B4] Snounou G White NJ The co-existence of *Plasmodium*: sidelights from falciparum and vivax malaria in Thailand Trends Parasitol 2004 20 333 339 10.1016/j.pt.2004.05.004 15193565

[B5] Putaporntip C Hongsrimuang T Seethamchai S Kobasa T Limkittikul K Cui L Jongwutiwes S Differential prevalence of *Plasmodium *infections and cryptic *Plasmodium knowlesi *malaria in humans in Thailand J Infect Dis 2009 199 1143 1150 10.1086/597414 19284284 PMC8817623

[B6] Mharakurwa S Simoloka C Thuma PE Shiff CJ Sullivan DJ PCR detection of *Plasmodium falciparum *in human urine and saliva samples Malar J 2006 5 103 10.1186/1475-2875-5-103 17092335 PMC1654175

[B7] Nwakanma DC Gomez-Escobar N Walther M Crozier S Dubovsky F Malkin E Locke E Conway DJ Quantitative detection of *Plasmodium falciparum *DNA in saliva blood and urine J Infect Dis 2009 199 1567 1574 10.1086/598856 19432544

[B8] Sutherland CJ Hallett R Detecting malaria parasites outside the blood J Infect Dis 2009 199 1561 1563 10.1086/598857 19432543

[B9] Thimasarn K Jatapadma S Vijaykadga S Sirichaisinthop J Wongsrichanalai C Epidemiology of malaria in Thailand J Travel Med 1995 2 59 65 10.1111/j.1708-8305.1995.tb00627.x 9815363

[B10] Zhou G Sirichaisinthop J Sattabongkot J Jones J Bjørnstad ON Yan G Cui L Spatio-temporal distribution of *Plasmodium falciparum *and *P vivax *malaria in Thailand Am J Trop Med Hyg 2005 72 256 262 15772317

[B11] World Health Organization Severe falciparum malaria Trans R Soc Trop Med Hyg 2000 94 S1 S90 11103309

[B12] Kain KC Brown AE Mirabelli L Webster HK Detection of *Plasmodium vivax *by polymerase chain reaction in a field study J Infect Dis 1993 168 1323 1326 8228373 10.1093/infdis/168.5.1323

[B13] Landis JR Koch GG An application of hierarchical kappa-type statistics in the assessment of majority agreement among multiple observers Biometrics 1977 33 363 374 10.2307/2529786 884196

[B14] Murray CK Gasser RA Magill AJ Miller RS Update on rapid diagnostic testing for malaria Clin Microbiol Rev 2008 21 97 110 10.1128/CMR.00035-07 18202438 PMC2223842

